# Supplementary material for: Causal Relationship between Aging and Anorexia Nervosa: A White-Matter-Microstructure-Mediated Mendelian Randomization Analysis
Source: Biomedicines. 2024 Aug 16;12(8):1874. doi: 10.3390/biomedicines12081874 (PMC11351342; doi:10.3390/biomedicines12081874)
Supplement: Supplementary file 1 [file biomedicines-12-01874-s001.zip › Supplementary Figure/Figure S9/Leaveoneout plot of MR analysis for ubm-b-1605 to AN.pdf]

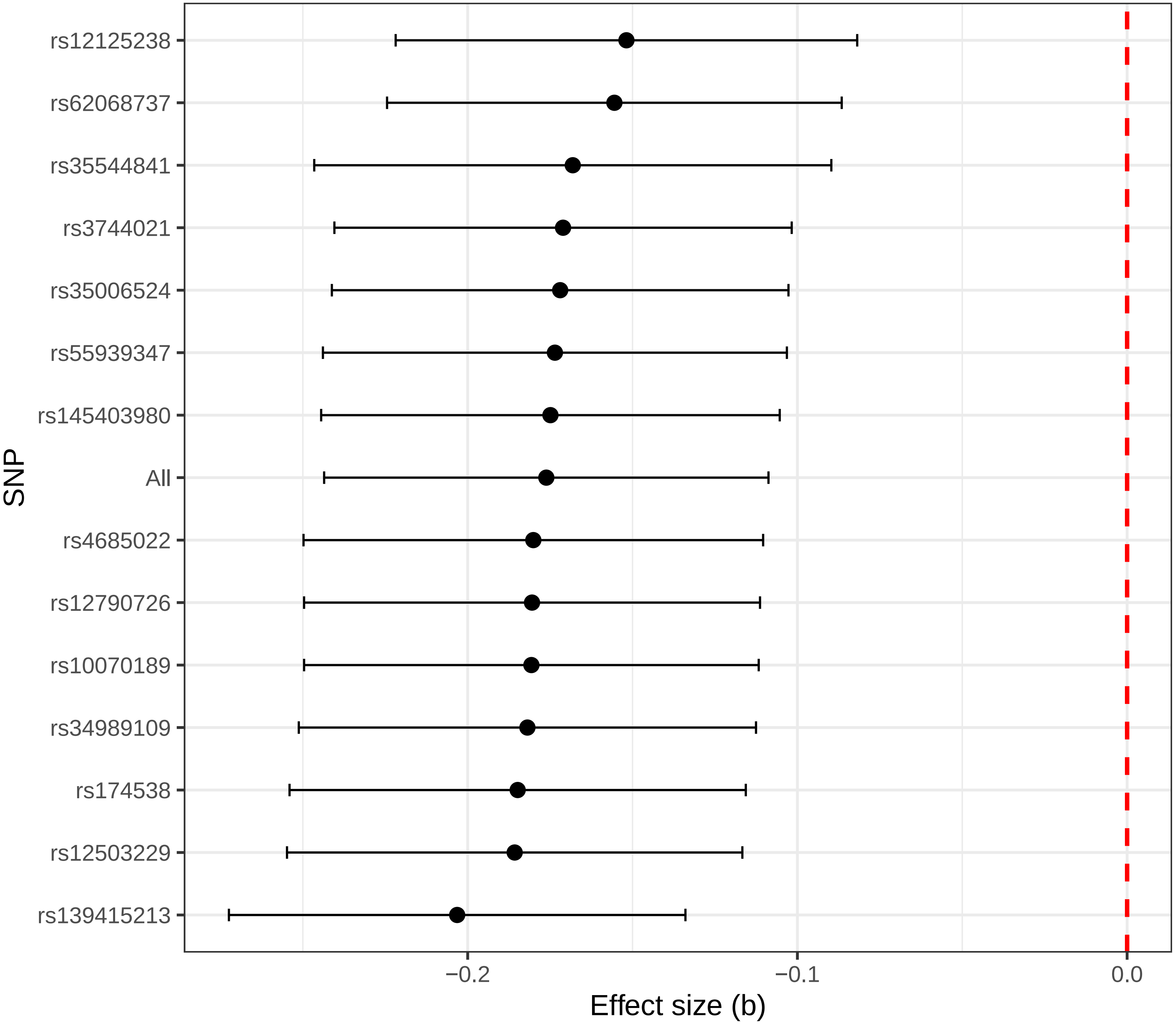
rs12125238
rs62068737
rs35544841
rs3744021
rs35006524
rs55939347
rs145403980
All
rs4685022
rs12790726
rs10070189
rs34989109
rs174538
rs12503229
rs139415213
SNP
−0.2
−0.1
0.0
Effect size (b)
